# Localised pericardial effusion mimicking anterior myocardial infarction following coronary angiography

**DOI:** 10.5830/CVJA-2015-086

**Published:** 2016

**Authors:** Aynur Acibuca, Demet Menekse Gerede, Veysel Ozgur Baris, Mustafa Kilickap

**Affiliations:** Department of Cardiology, Ankara University School of Medicine, Ankara, Turkey; Department of Cardiology, Ankara University School of Medicine, Ankara, Turkey; Department of Cardiology, Ankara University School of Medicine, Ankara, Turkey; Department of Cardiology, Ankara University School of Medicine, Ankara, Turkey

**Keywords:** regional pericarditis, myocardial infarction, acute stent thrombosis, located pericardial effusion

## Abstract

The diagnosis of pericarditis is important, especially in patients assumed to have acute coronary syndrome. Distinguishing these two conditions is vital but not always easy. Accurate diagnosis is essential to provide appropriate treatment as soon as possible and to avoid inappropriate invasive procedures. By highlighting this distinction, we report a case of pericarditis that occurred after percutaneous coronary intervention and mimicked acute coronary syndrome.

## Abstract

Regional pericarditis has been described but remains a relatively unknown and under-diagnosed condition. There are no electrocardiography (ECG) criteria to diagnose regional pericarditis and only a few studies have investigated regional pericarditis. Although regional pericarditis is usually observed in patients following myocardial infarction (MI), it has also been reported in other conditions.^1^,^2^ We present a case of regional pericarditis with electrocardiographic features mimicking anterior MI.

## Case report

**Fig. 1.**
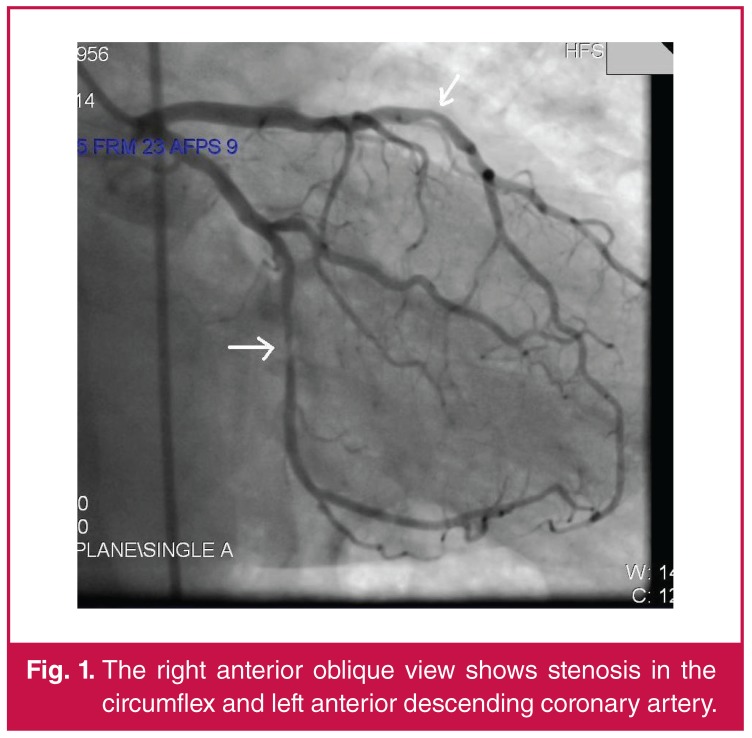
The right anterior oblique view shows stenosis in the circumflex and left anterior descending coronary artery

**Fig. 2.**
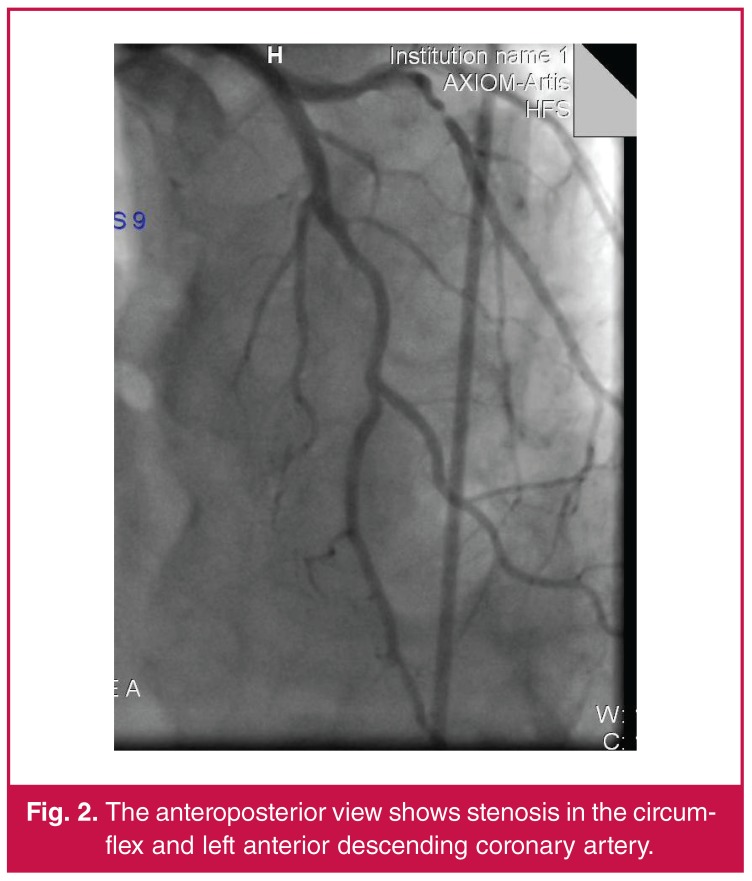
The anteroposterior view shows stenosis in the circumflex and left anterior descending coronary artery.

A 58-year-old male smoker presented with a one-month history of exercise chest pain. His exercise ECG was borderline normal, so coronary angiography (CAG) was performed. The CAG revealed severe stenosis in the circumflex and right coronary artery (RCA). Borderline severe stenosis was also detected in the left anterior descending (LAD) coronary artery (Fig. 1, Fig. 2). The fractional flow reserve value was 0.92.

Two stents were implanted in the RCA and circumflex artery, one after the other. Immediately after the procedure, the patient developed chest pain. An emergency CAG did not identify any culprit lesion.

**Fig. 3.**
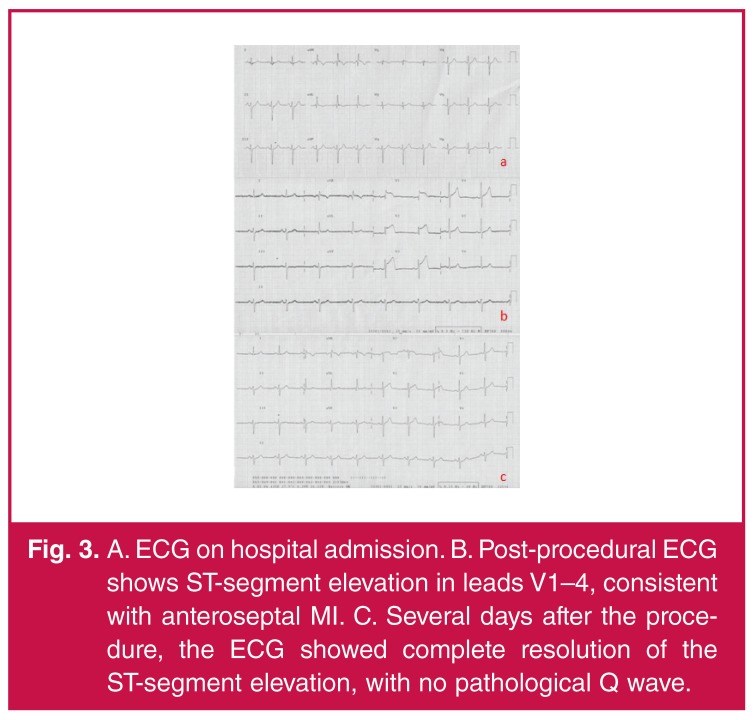
A. ECG on hospital admission. B. Post-procedural ECG shows ST-segment elevation in leads V1–4, consistent with anteroseptal MI. C. Several days after the procedure, the ECG showed complete resolution of the ST-segment elevation, with no pathological Q wave.

Half an hour after the second CAG, the patient complained of severe chest pain. An ECG revealed ST-segment elevation in leads V1–4, consistent with anteroseptal MI (Fig. 3B), which had not been there before (Fig. 3A). The patient was taken immediately to the catheterisation unit.

There was no occlusion in the implanted stents but the stent in the RCA was under-expanded. Dilatation was performed, however, the patient continued to experience chest pain. Therefore, a stent was implanted for LAD stenosis. Initially, the chest pain decreased but then increased again. A second stent was deployed in the suspected dissection region in the LAD.

**Fig. 4.**
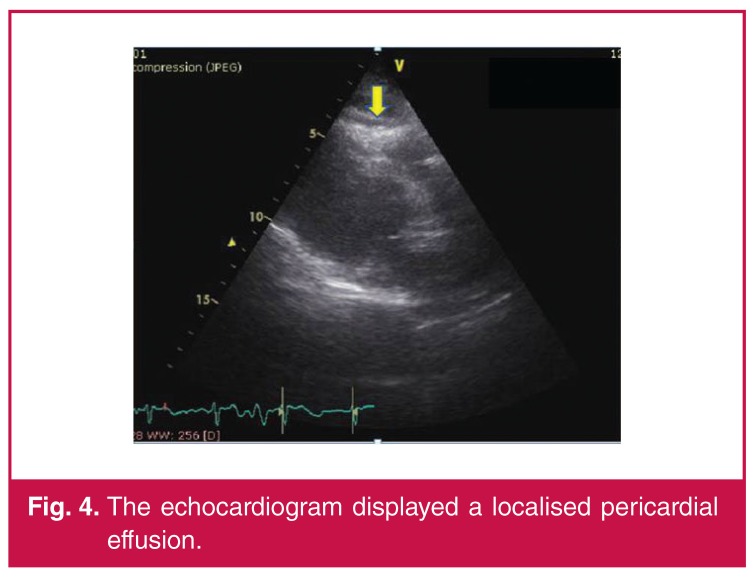
The echocardiogram displayed a localised pericardial effusion.

Echocardiography confirmed a structurally normal heart with no obvious regional wall abnormality. An echocardiogram revealed a localised apical pericardial effusion (Fig. 4).

**Fig. 5.**
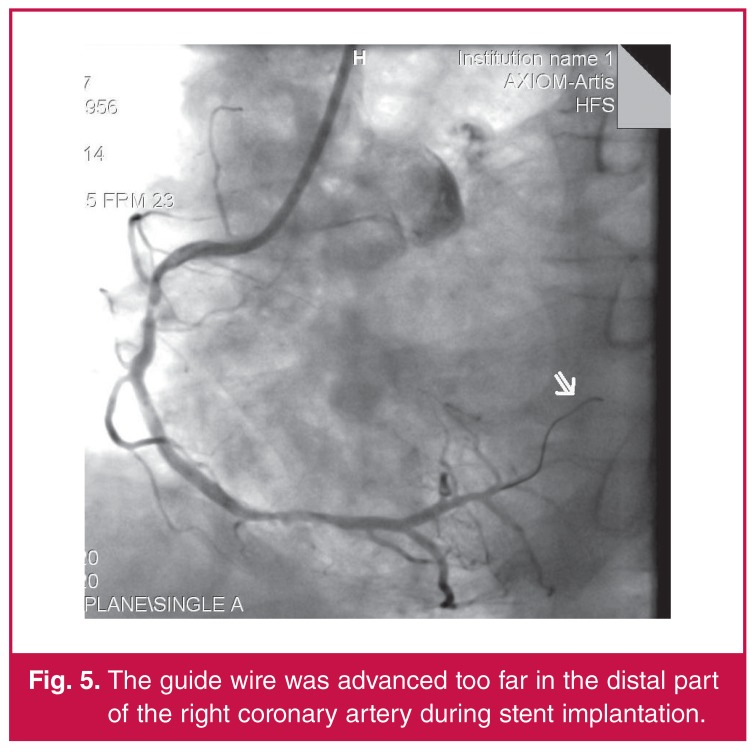
The guide wire was advanced too far in the distal part of the right coronary artery during stent implantation.

The patient’s chest pain remained constant for several hours, without any recurrence of elevated cardiac enzymes. His chest pain was attributed to local pericardial irritation due to coronary perforation by the guide wire during implantation of the stent (Fig. 5).

Several days after the procedure, the ECG showed complete resolution of the ST-segment elevation, with no pathological Q wave (Fig. 3C). Given the combination of symptoms, ECG changes and echocardiographic findings, a diagnosis of regional pericarditis was made, despite the absence of a pericardial rub, which is fleeting in nature.

## Discussion

It is important for the clinician to differentiate acute MI/acute stent thrombosis from pericarditis, which is a rare complication of percutaneous coronary intervention. It can be difficult to distinguish regional pericarditis from myocardial ischaemia with ECG.

Echocardiography can be very useful in excluding regional wall motion abnormalities and identifying pericardial effusion, especially in atypical presentations of pericarditis. However, in the acute setting, prompt differentiation of pericarditis from myocardial injury by ECG remains of paramount importance to avoid a delay in reperfusion.

Earlier reports confirm that it is frequently difficult to differentiate between acute pericarditis and coronary occlusion.^3^,^4^ The problem appears to be further confounded when pericarditis is regional, with electrocardiographic features nearly indistinguishable from localised MI, which could lead to the incorrect treatment.^5^

In this case, coronary perforation by the tip of the guide wire most likely caused injury to the local pericardium,^6^ as evidenced by the anterior injury pattern that developed on the patient’s ECG, mimicking MI. The complete resolution of the patient’s ECG abnormalities, the absence of wall motion abnormalities, and the lack of elevation of troponin I levels all support the diagnosis of regional pericarditis.

## Conclusion

Pericarditis is a common condition with clinical and electrocardiographic features that can mimic acute coronary syndromes. The subtle differences between the two conditions are often overlooked due to the fear of missing the more serious diagnosis of acute coronary syndrome and the window for timely reperfusion.
